# Computational studies for pre-evaluation of pharmacological profile of gut microbiota-produced gliclazide metabolites

**DOI:** 10.3389/fphar.2024.1492284

**Published:** 2024-12-03

**Authors:** Maja Đanić, Nebojša Pavlović, Dragana Zaklan, Bojan Stanimirov, Slavica Lazarević, Hani Al-Salami, Momir Mikov

**Affiliations:** ^1^ Department of Pharmacology, Toxicology and Clinical Pharmacology, Faculty of Medicine, University of Novi Sad, Novi Sad, Serbia; ^2^ Department of Pharmacy, Faculty of Medicine, University of Novi Sad, Novi Sad, Serbia; ^3^ Department of Biochemistry, Faculty of Medicine, University of Novi Sad, Novi Sad, Serbia; ^4^ The Biotechnology and Drug Development Research Laboratory, Curtin Medical School and Curtin Health Innovation Research Institute, Curtin University, Perth, WA, Australia

**Keywords:** drug metabolites, pharmacomicrobiomics, gut microbiota, diabetes mellitus, drug metabolism

## Abstract

**Background:**

Gliclazide, a second-generation sulfonylurea derivative still widely used as a second-line treatment for type 2 diabetes mellitus, is well known to be subject to interindividual differences in bioavailability, leading to variations in therapeutic responses among patients. Distinct gut microbiota profiles among individuals are one of the most crucial yet commonly overlooked factors contributing to the variable bioavailability of numerous drugs. In light of the shift towards a more patient-centered approach in diabetes treatment, this study aimed to conduct a pharmacoinformatic analysis of gliclazide metabolites produced by gut microbiota and assess their docking potential with the SUR1 receptor to identify compounds with improved pharmacological profiles compared to the parent drug.

**Methods:**

Ten potential gliclazide metabolites produced by the gut microbiota were screened for their pharmacological properties. Molecular docking analysis regarding SUR1 receptor was performed using Molegro Virtual Docker software. Drug-likeness properties were evaluated using DruLiTo software. Subsequently, the physicochemical and pharmacokinetic properties of gliclazide and its metabolites were determined by using VolSurf+ software package.

**Results:**

All studied metabolites exhibited better intrinsic solubility than gliclazide, which is of interest, considering that solubility is a limiting factor for its bioavailability. Based on the values of investigated molecular descriptors, hydroxylated metabolites M1-M6 showed the most pronounced polar and hydrophilic properties, which could significantly contribute to their *in vivo* solubility. Additionally, docking analysis revealed that four hydroxyl-metabolites (M1, M3, M4, and M5), although having a slightly poorer permeability through the Caco-2 cells compared to gliclazide, showed the highest binding affinity to the SUR1 receptor and exhibited the most suitable pharmacological properties.

**Conclusion:**

*In silico* study revealed that hydroxylated gut microbiota-produced gliclazide metabolites should be further investigated as potential drug candidates with improved characteristics compared to parent drug. Moreover, their part in the therapeutic effects of gliclazide should be additionally studied *in vivo*, in order to elucidate the role of gut microbiota in gliclazide pharmacology, namely from the perspective of personalized medicine.

## 1 Introduction

Diabetes mellitus (DM) is a long-term metabolic disorder and a considerable global health concern that majorly impacts the life quality of more than 537 million people - a number that has been predicted to rise to 643 million by 2030. According to the 2021 IDF Diabetes Atlas, 6.7 million deaths and at least $966 billion in health expenditure can be ascribed to diabetes (International Diabetes Federation, 2021; Su et al., 2023; Parker et al., 2024). Type 2 diabetes mellitus (T2DM) accounts for 90% of DM-associated cases worldwide.

Notably, over the years, T2DM treatment strategies have undergone a profound transformation and the paradigm has shifted from a glucose-centered to a more patient-centered approach which takes into consideration specific attributes and needs of each individual, while attempting not to overlook pharmaco-economic aspects of the procedure (Chong et al., 2024). Despite the evident change in the landscape caused by the launch of novel oral antidiabetics, sulfonylurea derivatives are still widely accepted as a second-line treatment after metformin, being advantageous in terms of several decades long clinical experience, effectiveness and safety when reasonably used, as well as more favorable cost (Scheen, 2021).

Gliclazide is a second-generation sulfonylurea derivative used in the treatment of T2DM that exerts its effects by selectively binding to the sulfonylurea receptor 1 (SUR1) on the membrane of pancreatic β-cells, triggering the exocytosis of insulin granules upon the activation (Singh and Singh, 2016). In addition to a well-known role of gliclazide in T2DM, Mikov et al. have extensively studied and documented the potential role of gliclazide in type 1 diabetes mellitus (T1DM), highlighting the link between diabetes, disruptions in gut microbiota composition, and bile acid secretion (Mikov et al., 2018). Chemically, gliclazide is a weak acid and a small lipophilic molecule containing three chemical groups: an aromatic ring, a sulfonylurea group, and an azabicyclic ring. According to the Biopharmaceutics Classification System (BCS), gliclazide belongs to Class II drugs, characterized by low water solubility and high permeability. For such active pharmaceutical ingredients, the dissolution rate is the limiting factor for bioavailability (Biswal et al., 2009). Gliclazide is extensively metabolized in the liver, resulting in the formation of seven metabolites through hydroxylation, N-oxidation, and oxidation reactions, none of which have been shown to possess hypoglycemic activity (Oida et al., 1985). Of interest, significant interindividual differences in the bioavailability of gliclazide have been observed, leading to substantial variations in therapeutic responses among patients (Holstein et al., 2011). Potential explanation for these variabilities could be drug metabolism influenced by gut microbiota, one of the most crucial but commonly overlooked factors for the variable bioavailability and unpredicted therapeutic outcome of numerous drugs (Stojančević et al., 2014; Wilson and Nicholson, 2017).

The gut microbiota represents a complex, diverse and dynamic community of microorganisms, mostly obligate anaerobic bacteria, that exist in a symbiotic relationship with the host and play a crucial role in various physiological processes, such as digestion, synthesis of essential nutrients, and interaction with both immune and nervous systems. The microbiota composition is unique to each individual, forming a distinctive bacterial “fingerprint” that plays a key role in human health and the diseases predisposition. Additionally, this one-of-a-kind microbiota composition can influence therapeutic responses to drugs by participating in their biotransformation, affecting drug transport, and modifying various gastrointestinal properties (Djanic et al., 2016; Zhang et al., 2021; Dikeocha et al., 2022; Wallenborn and Vonaesch, 2022). Apart from indigenous intestinal microbiota, probiotic supplements can also impact this process, with potentially significant implications for xenobiotics pharmacology and toxicology (Purdel et al., 2023). Orally administered drugs, particularly those with low solubility and/or permeability reach the lower portions of the gastrointestinal tract (GIT) where bacteria are most abundant. Subsequently, they become exposed to direct interactions with the microbiota expressing a broad range of enzymes involved in decarboxylation, dehydroxylation, dealkylation, acetylation and deacetylation, denitration, N-demethylation, dehalogenation, deamination, thiazole ring opening, as well as the metabolism of glutathione-conjugated xenobiotics excreted in bile. This modulation may result in the production of physiologically active, inactive, and even toxic drug metabolites (Sousa et al., 2008; Stojančević et al., 2014; Đanić and Mikov, 2020). The involvement of gut microbiota in drug metabolism has been documented for drugs like irinotecan, digoxin, lactulose, sulfasalazine, paracetamol, simvastatin and metformin (Bojic et al., 2014; Sun et al., 2019; Liu et al., 2022; Đanić et al., 2023). Furthermore, gut microbiota may potentially synthesize a variety of other structurally distinct drug metabolites. These compounds are hoped to exhibit bioactivity and promising pharmacological effects on hosts. Interdisciplinary research effort is made to elucidate the therapeutic potential of such microbial metabolites, which, for the most part, still remain unknown (Liu et al., 2022).

In an *in vivo* study, our team has shown that probiotic pre-treatment reduces bioavailability of gliclazide in healthy rats (Al-Salami et al., 2008). As stated in discussion of that study, decreased bioavailability may stem from bacterial degradation, so it was suggested to explore in more detail the mechanisms behind these effects. In addition, our previous research demonstrated that during incubation of gliclazide with certain probiotic bacteria, the total amount of gliclazide decreased over time, reaching 70% of the initial value after 24 h, indicating that part of the drug may be metabolized by bacterial enzymes in the intestinal tract (Ðanić et al., 2019).

Investigation of gut microbiota-mediated metabolism of gliclazide is not only of immense importance in the context of personalized diabetes treatment, but also in elucidating the mechanisms of gliclazide’s action in T1DM, as well as in the development of novel chemical entities with improved pharmacological properties compared to gliclazide itself. In addition, these metabolites might contribute to the therapeutic effect of gliclazide in T2DM patients (Zhang and Tang, 2018).

In recent years, information and communication technologies have substantially advanced and changed the landscape of drug research and development. *In silico* methods and computational techniques have become integral to every aspect of it, from identifying drug targets and discovering potential candidates, to optimizing leads and forecasting safety concerns, all of which immensely contribute to increasing the success rates and lowering overall costs of drug discovery (Arrué et al., 2022; Iwata, 2023). Moreover, the significance of computational modeling and simulation has been acknowledged and encouraged by the regulatory agencies within the drug regulatory process (Jose et al., 2022). The growing interest in microbial metabolism and the recognition of the importance of gut microbiota-host cross-talk in human health and disease call for the application of *in silico* methods, which enable rapid screening of a large number of compounds and offer insight into the metabolic pathways involved. Thus, *in silico* research allows for the more successful identification and selection of novel potential therapeutic agents with favorable metabolic profiles (Kolodnitsky et al., 2023). Based on our aforementioned *in vivo* and *in vitro* findings (Al-Salami et al., 2008; Ðanić et al., 2019), this study aimed to perform a pharmacoinformatic analysis of the putative pharmacological profile of gliclazide metabolites produced by gut microbiota and to perform a docking analysis of gliclazide and its metabolites with the SUR1 receptor, which, to the best of our knowledge, have not been previously reported. By leveraging computational methods and pharmacological modeling, this research sought to uncover novel drug-like compounds with potentially improved therapeutic properties, thereby offering valuable insights for the development of more effective treatments for diabetes. The discovery of the gut microbiota implication in the therapeutic response can provide novel knowledge on the underlying molecular mechanisms of T2DM patients’ response to therapy. This insight has the potential to drive advancements in T2DM therapy and to facilitate the creation of personalized treatment algorithms tailored to individual patient needs.

## 2 Materials and methods

### 2.1 Docking analysis of gliclazide and its metabolites towards the SUR1 receptor

#### 2.1.1 Protein and ligand preparation

The three-dimensional (3D) crystal structure of the SUR1 receptor in a complex with glibenclamide, which was recently determined using electron microscopy at a resolution of 4.11 Å (Wu et al., 2018), was retrieved from the RCSB Protein Data Bank in PDB format (PDB code: 5YKE) (Berman et al., 2000).

Potential biotransformation pathways of gliclazide under the influence of microbial enzymes were investigated using *in silico* methods with appropriate software packages in a previously published study (Ðanić et al., 2019) and accordingly, ten metabolites were predicted and used for current analysis. The structures of gliclazide and its metabolites were drawn using ChemDraw 16.0 software and saved in two-dimensional (2D) mol format. The conversion to corresponding 3D structures with optimized geometries in mol2 format was performed using the MM2 method in Chem3D 16.0 software.

#### 2.1.2 Molecular docking analysis

Docking studies were conducted using Molegro Virtual Docker (MVD) software, version 6.0 (Thomsen and Christensen, 2006). MVD is widely employed in drug development processes due to its high reliability, based on the application of differential evolution algorithms that consider both the intermolecular interaction energy between the protein and ligand, and the ligand’s intramolecular interaction energy. The scoring function for evaluating the energetically most favorable orientations of the ligand during interaction with the target macromolecule is based on a piecewise linear function that includes electrostatic interactions and hydrogen bonding (Pradeep Singh and Kumar Konwar, 2014).

Gliclazide and its metabolites in mol2 format, and the SUR1 receptor in pdb format, were imported into the MVD software. All solvent molecules were removed from the protein structure. The validation of the docking protocol was conducted by “redocking” the co-crystalized ligand (glibenclamide) in the structure of SUR1. The root mean square distance (RMSD) value of the docked glibenclamide was lower than 2 Å, confirming that docked and preexisting co-crystallized glibenclamide were able to interact with the SUR1 active site in a similar manner.

Initially, potential ligand binding sites on the SUR1 receptor were predicted, and the cavity with a surface area 5445.12 Å^2^ and a volume of 3721.22 Å³ containing the co-crystallized glibenclamide was selected as the active site for further docking analyses. Different ligand orientations were explored and ranked based on binding energy scores. Grid-based MolDock score (GRID) function with a grid resolution of 0.30 Å was used for the calculation of binding energies and to obtain MolDock scores as indicators of ligand affinity towards the receptor. The binding site on the receptor was defined as a sphere encompassing all protein atoms within a radius of 20 Å from the co-crystallized ligand. Each ligand was docked to the protein 10 times with a maximum of 1,500 iterations to obtain reliable binding energy values expressed in kcal/mol (MolDock score). A lower MolDock score indicates a more stable complex formed between the ligand and the receptor. The conformation with the lowest binding energy was selected for further analysis of the interactions of gliclazide and its metabolites with the SUR1 receptor.

### 2.2 Determination of molecular descriptors in assessing the pharmacological profile of gliclazide metabolites

In order to examine the potential application of identified gliclazide metabolites as therapeutic substances, as well as to compare their properties with gliclazide itself, drug-likeness analyses were conducted. These analyses, based on predefined criteria and calculated molecular descriptors, determine whether a substance is a candidate for further investigation as a potential drug.

An *in silico* preliminary screening of drug-likeness properties of gliclazide and its metabolites was performed using DruLiTo software. In addition to Lipinski’s rule as the most commonly used filter for assessing drug-likeness properties of chemical entities, Veber’s filter, Ghose’s filter, and the Quantitative Estimate of Drug-likeness (QED) were also applied (Di and Kerns, 2003).

Molecular descriptors describing the physicochemical and pharmacokinetic properties of the compounds were determined using the software package VolSurf+, version 1.0.4 (Molecular Discovery Ltd.). VolSurf+ utilizes advanced computational procedures that convert information present in 3D maps of interaction energies between molecules and known chemical probes into numerical descriptors optimized for AMDE models, which are straightforward for understanding and interpretation (Cruciani et al., 2000). A detailed list of computed molecular descriptors, along with explanations for each descriptor, is provided in Table 1.

**TABLE 1:** Molecular descriptors used for assessing the pharmacological profile of gliclazide metabolites.

Descriptor label	Descriptor name	Explanation
A	Amphiphilic moment	Length of the vector from the center of the hydrophobic domain to the center of the hydrophilic domain, indicating the compound’s permeation ability
LogP	LogP in octanol/water	Negative decimal logarithm of the partition coefficient in octanol/water system
PSA	Polar Surface Area	Sum of polar surface areas of molecules in Å^2^
HSA	Hydrophobic Surface Area	Sum of non-polar surface areas of molecules in Å^2^
PSAR	The ratio of polar and total surface area	Percentage of polar surface areas in the entire molecule
PHSAR	The ratio of polar and hydrophobic surface area	Ratio of total polar surface areas to non-polar surface areas of molecules
SOLY	Intrinsic solubility	Decimal logarithm of solubility of the unionized or neutral form of molecules in mol/L at 25 °C
PB	Protein binding	Percentage of plasma protein binding
VD	Volume of distribution	Negative decimal logarithm of the volume of distribution expressed in L/kg
CACO2	Caco-2 permeability	Qualitative indicator of penetration through Caco-2 intestinal cells
LgBB	Distribution through blood brain barrier	Logarithm of distribution across the blood-brain barrier; values less than −0.5 indicate poor permeability, while values greater than 0.5 indicate high permeability
MetStab	Metabolic stability	Percentage of a drug remaining unchanged after incubation with CYP3A4; values above 50 indicate stable behavior
HTSFlag	High Throughput Screening Flag	A value of 0 indicates potential, and a value of 1 indicates impossibility of using the compound in *in silico* screening

In order to assess the suitability of applied *in silico* methods for the investigated set of compounds, a high-throughput screening flag (HTSFlag) parameter was determined, and this descriptor had a value of 0 for all tested compounds, confirming their suitability for *in silico* studies.

## 3 Results

### 3.1 Structure of ligands

**FIGURE 1:**
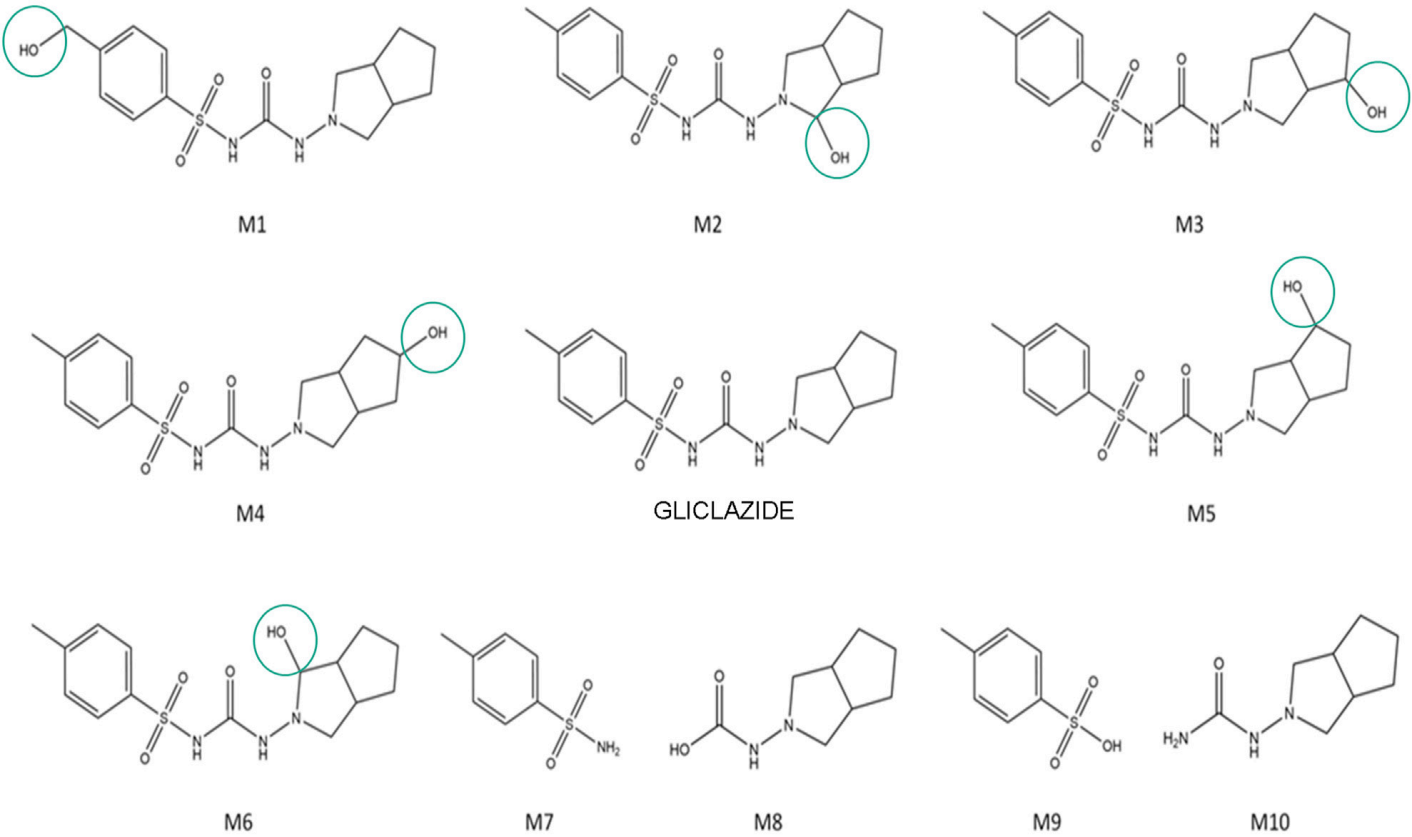
Proposed structures of gliclazide and its metabolites.

The putative structures of gliclazide metabolites, along with gliclazide itself, are shown in Figure 1. Metabolites M1-M6 are hydroxylated metabolites of gliclazide, while M7-M10 are products of microbial C–N and S–N bond hydrolysis.

### 3.2 Docking analysis of gliclazide and its metabolites towards the SUR1 receptor

The results from the docking analysis reveal the binding affinities of gliclazide metabolites to SUR1 receptors on pancreatic β cells. Table 2 presents the obtained MolDock Score results, which indicate the binding energy of each investigated gliclazide metabolite to the receptor protein, expressed in kcal/mol (Table 2).

**TABLE 2:** Results of Docking analysis.

Ligand	MolDock Score [kcal/mol]	Hbond [kcal/mol]
Gliclazide	−116.34	0
M1	−119.57	−1.58
M2	−121.30	−0.64
M3	−126.29	−1.13
M4	−125.99	−1.81
M5	−129.15	−4.55
M6	−125.23	−1.97
M7	−72.57	−0.80
M8	−95.96	0
M9	−78.48	0
M10	−89.86	0

**FIGURE 2:**
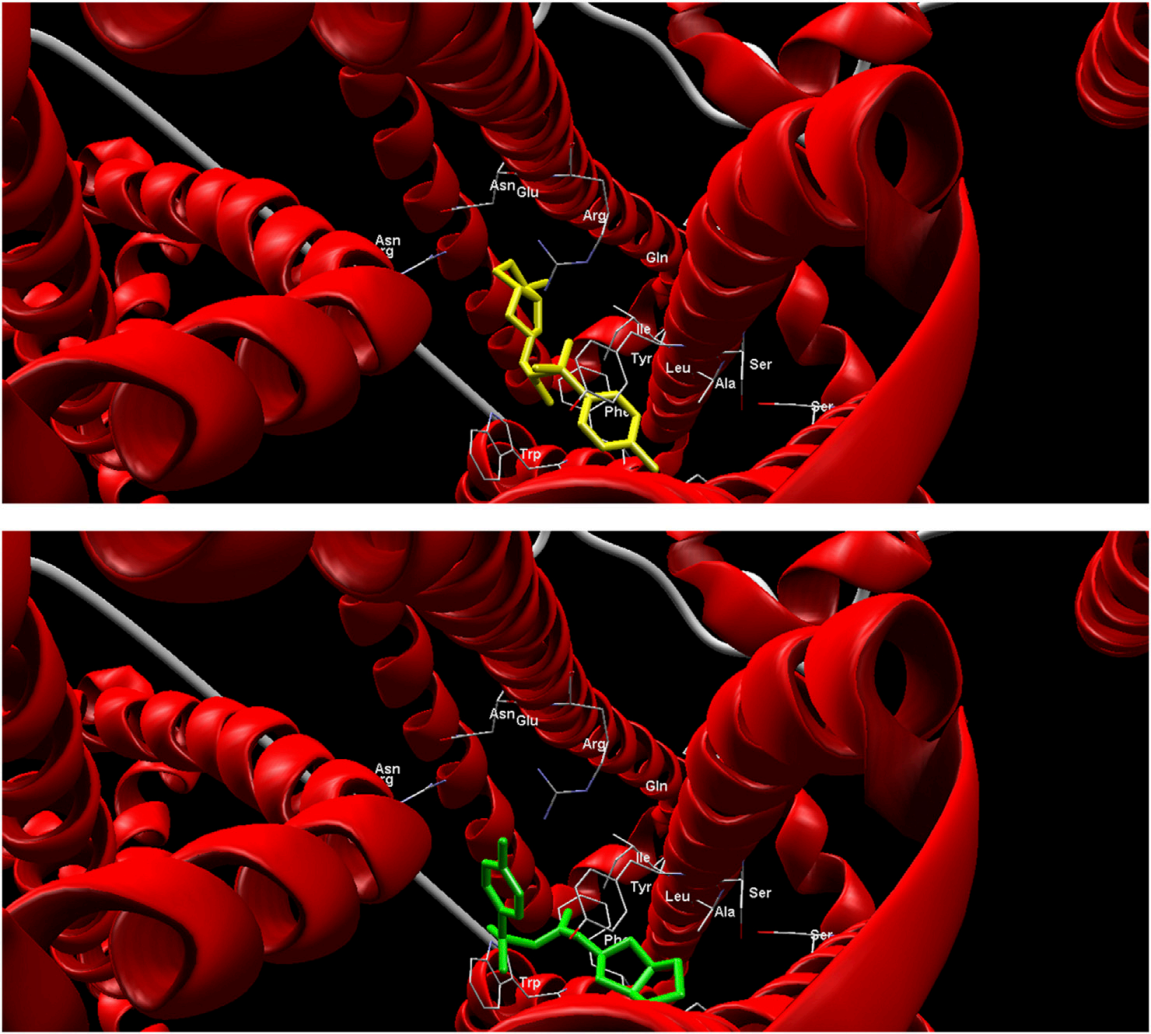
Binding mode of gliclazide metabolite M5 in the active site SUR 1 receptor.

Compared to the gliclazide molecule, which has a binding energy of −116.34 kcal/mol, five metabolites (M1-M6) have lower binding energies, ranging from −119.57 kcal/mol to −129.15 kcal/mol, and four metabolites (M7-M10) have higher binding energies, ranging from −72.57 kcal/mol to −95.96 kcal/mol. The metabolite M5 exhibits the lowest MolDock Score of −129.15 kcal/mol, indicating that the M5-SUR1 complex is the most stable (Figure 2). Accordingly, this metabolite shows the highest contribution of hydrogen bonds to complex stabilization (−4.55 kcal/mol). On the other hand, metabolite M7 has the highest MolDock Score of −72.57 kcal/mol, suggesting that its complex with the receptor is the least stable.

### 3.3 Pharmacoinformatics analysis of the pharmacological profile of gliclazide metabolites

Results obtained using DruLiTo program indicate the drug-likeness properties of gliclazide metabolites, specifically identifying which metabolites from the mentioned list fulfill certain criteria to be included in further investigations and potentially become therapeutic agents. These results are summarized in Table 3.

**TABLE 3:** Evaluation of drug-likeness properties of gliclazide metabolites.

Drug-likeness filter	Rules that must be met within these filters*	The number of analogues that violate the rules of the filter
Lipinski rule of five	M_w_ ≤ 500, logP ≤5, HBA ≤10, HBD ≤5	11
Ghose Filter	M_w_: 160–480, MR: 40-130, logP: -0.4-5.6, NoA: 20-70	9
Webber Filter	Number of rotatable bonds ≤10, PSA ≤140	11
QED Filter	QED ≥0.5	11

MW-molecular mass, HBA-hydrogen acceptors, HBD-hydrogen donors, MR-molar refractivity, NoA - number of atoms, PSA-polar surface, QED-mathematical models that quantitatively evaluate drug-likeness (includes MW, AlogP, HBA, HBD, rotating bonds, aromatic bonds; PSA, number of structural alerts).

Based on results shown in Table 3, it is evident that all compounds (all 10 metabolites M1–M10 along with gliclazide itself) meet Lipinski’s rule of five, the Webber filter, and the QED Filter criteria. Ghose Filter was not satisfied only for metabolites M7-M10. This filter defines limitations as follows: the calculated logP is between −0.4 and 5.6, the molecular weight ranges from 160 to 480 g/mol, the molar refractivity is between 40 and 130, and the total number of atoms falls between 20 and 70 (Klünemann et al., 2014). Metabolites M7–M10 do not meet this requirement due to a smaller number of atoms in their structure. Since the criteria of Lipinski and the most advanced mathematical models quantitatively assessing drug-likeness (QED) are met, all metabolites are included in further investigations. Nevertheless, metabolites M7-M10 should be considered with caution due to their failure to meet the Ghose filter criteria.

Molecular descriptors relevant for evaluating the pharmacological profiles of the investigated compounds are presented in Table 4.

**TABLE 4:** Molecular descriptors for gliclazide and metabolites obtained by VolSurf+ program.

Compound	logP	PSA	HSA	PSAR	PHSAR	SOLY	PB	VD	CACO2	LgBB	MetStab	HTSflag
Gliclazide	1.66	86.53	415.18	0.17	0.21	−3.25	63.53	−0.48	0.32	−0.62	74.58	0.00
M1	0.41	106.76	403.17	0.21	0.26	−2.71	47.29	−0.52	−0.35	−1.21	93.22	0.00
M2	1.28	106.76	393.70	0.21	0.27	−3.03	48.81	−0.45	−0.07	−0.84	86.85	0.00
M3	0.66	106.76	395.56	0.21	0.27	−2.70	52.91	−0.39	−0.18	−0.95	90.77	0.00
M4	0.66	106.76	391.27	0.21	0.27	−2.77	51.84	−0.39	−0.20	−0.97	89.76	0.00
M5	0.66	106.76	404.34	0.21	0.26	−2.83	50.27	−0.39	−0.20	−0.97	87.87	0.00
M6	1.28	106.76	392.30	0.21	0.27	−3.24	49.18	−0.41	0.03	−0.97	82.26	0.00
M7	0.93	68.18	225.97	0.23	0.30	−1.37	49.83	0.05	0.85	0.13	100.00	0.00
M8	0.64	52.57	251.12	0.17	0.21	−1.30	32.40	−0.12	0.74	−0.05	100.00	0.00
M9	−0.09	62.39	227.47	0.22	0.27	−0.20	42.64	−0.19	0.22	−1.06	100.00	0.00
M10	0.00	58.36	249.46	0.19	0.23	−1.06	34.95	−0.01	0.72	−0.25	100.00	0.00

Based on the results obtained, it can be observed that all metabolites exhibit lower partition coefficient (logP) values than gliclazide itself, with logP 1.66. Consequently, all metabolites show a higher proportion of hydrophilic character compared to gliclazide. The metabolites M1–M6 have the highest polar surface area (PSA) with a value of 106.76 Å^2^, while M8 has the lowest with 52.57 Å^2^. Gliclazide itself has the highest hydrophobic surface area (HSA) at 415.18 Å^2^; all metabolites have lower values, with M7 having the lowest at 225.97 Å^2^. All metabolites exhibit a higher PSAR value (ratio of polar surface area to total surface area) compared to gliclazide, except for M8, which has an equal value to gliclazide at 0.17. The same trend is observed for the polar to hydrophobic surface area ratio (PHSAR), where all metabolites have higher values than gliclazide, except for M8, which has an equal value at 0.21.

Further examining the SOLY descriptor, all tested metabolites demonstrate better solubility of their unionized or neutral form compared to gliclazide compared to gliclazide (−3.25), with metabolite M9 (−0.20) showing the greatest solubility. Among the hydroxylated metabolites (M1-M6), M1, M3, M4, and M5 show slightly better solubility compared to gliclazide and metabolites M2 and M6.

Regarding pharmacokinetic parameters, gliclazide itself shows the highest plasma protein binding (PB) among all obtained metabolites, with a value of 63.53%. However, besides gliclazide, metabolites M3, M4, and M5 also show good binding with values of 52.91%, 51.84%, and 50.27%, respectively. All metabolites, except M1, have a higher volume of distribution (VD) than gliclazide, ranging from −0.45 to 0.05, with M7 having the highest value (0.05). Metabolites M7, M8, and M10 exhibit the best permeability through Caco-2 cells with values of 0.85, 0.74, and 0.72, respectively, while gliclazide itself has a value of 0.32. Metabolites M1-M5 show lower values of this descriptor ranging from −0.35 to −0.07. Values of LgBB below −0.50 indicate poor permeability across the blood-brain barrier, encompassing gliclazide and metabolites M1–M6. None of the tested metabolites possess a value above 0.50 indicating high permeability, with metabolite M7 being the closest at 0.13. Observing the MetStab descriptor values, all compounds have values above 50, indicating stability towards CYP3A4 enzymatic activity. Metabolites M7–M10 are the most stable with a value of 100.00.

## 4 Discussion

Sulfonylurea derivatives have been in clinical use for T2DM treatment since the 1960s. Although contemporary guideline recommendations vary between countries, noteworthy authorities still include sulfonylureas among the preferred therapeutic alternatives as second-line therapy for T2DM (Khunti et al., 2020). Notably, gliclazide is included in the World Health Organization Model List of Essential Medicines 23rd list (2023), along with empagliflozin and metformin, as oral hypoglycaemic agents (World Health Organization, 2023). Furthermore, gliclazide is advantageous over other sulfonylureas in terms of lower risk of severe hypoglycemia and cardiovascular mortality, weight neutrality, and antioxidative potential (Khunti et al., 2020; Sahin et al., 2024). Nevertheless, variability in gliclazide pharmacokinetics is well documented and may be attributed to its BCS class II properties, early dissolution in the stomach, extensive hepatic metabolism and physiological and formulation characteristics (Shaik et al., 2018; El-Ashmawy et al., 2021). Moreover, the role of personal gut microbiota may be one of the explanations for the variability in gliclazide’s bioavailability and pharmacokinetic profile, thus being the main focus of our study.

As previously mentioned, the rationale for this study is based on our previous *in vivo* and *in vitro* findings, which revealed that the glucose-lowering effect of gliclazide may be suscept to a wide interindividual variability, with bacterial degradation identified as one of the potential mechanisms (Al-Salami et al., 2008; Ðanić et al., 2019) so it was suggested to explore in more detail the mechanisms behind these effects. The formation of microbial metabolites of gliclazide might also be the explanation for the results obtained by Golocorbin-Kon et al. (Golocorbin-Kon et al., 2017) who demonstrated that the hypoglycaemic effects of orally administered gliclazide microcapsules in alloxan-induced T1DM rat model are not solely dependent on its serum concentrations, but rather on the gut metabolism, as suggested.

Based on these findings, this study was designed to determine the pharmacological profiles of putative gliclazide metabolites produced by gut microbiota activity using *in silico* methods and a pharmacoinformatic approach. In addition to the advancement of mass spectrometry technologies, *in silico* methods and the development of computational chemistry play a crucial role in analyzing the metabolic profile of compounds. These methods enable the prediction of drug behavior in the body based on physicochemical and pharmacokinetic properties, which previously required extensive experimental laboratory and clinical work (Di and Kerns, 2003; Ekins et al., 2007; Klünemann et al., 2014; Mikov et al., 2022). The application of *in silico* methods enables the rapid and efficient prediction of the physicochemical and pharmacokinetic properties of new compounds, significantly reducing the need for extensive experimental work. To evaluate the feasibility of further investigation of the proposed metabolic products of gliclazide, selected molecular descriptors related to physicochemical properties and pharmacokinetic behavior were determined *in silico*.

Potential biotransformation pathways of gliclazide by the enzymatic activity of investigated bacteria were predicted in a previously published studies conducted by Đanić et al. (Ðanić et al., 2019; Đanić et al., 2021), indicating that hydroxylation and hydrolytic reactions have the highest probability of occurring. Three of these metabolites are consistent with those identified in the hepatic metabolism of gliclazide (Oida et al., 1985) specifically metabolites formed by hydroxylation. However, our findings demonstrate that the additional metabolites may be formed by gut microbiota, specifically through hydrolytic reactions facilitated by the gut microbiota. The proposed hydrolytic reactions, involving C-N and S-N bond cleavage in the gliclazide molecule, are mediated by hydrolases from the EC 3.5 and EC 3.10 enzyme groups, respectively, and suggest the splitting of the molecule into two parts. Namely, unlike the liver, the gut microbiota is capable of catalyzing hydrolytic reactions, generating distinct metabolites. These hydrolytic reactions lead to the formation of unique metabolites specific to the activity of gut bacteria, adding complexity to the metabolic profile of gliclazide.

To compare the affinity of the proposed metabolites to the target SUR1 receptor with that of gliclazide itself, docking analyses were performed. The aim of this analysis was to assess how effectively the proposed metabolites can bind to the receptor, which is crucial for their pharmacological activity. Docking analyses revealed variations in affinity for the SUR1 receptor on pancreatic β-cells, with hydroxylated metabolites M1-M6 exhibiting higher affinity than gliclazide molecule itself. This increased affinity is due to additional stabilization of the ligand-receptor complex through hydrogen bonds originating from hydroxyl groups, which opens the possibility of achieving an additional hypoglycemic effect mediated by metabolites produced by gut microbiota activity. These hydroxylated gliclazide compounds could also be considered as potential therapeutic agents. Supporting this notion is the fact that some drugs have hydroxylated metabolites that exhibit stronger pharmacological effects than the parent drug, such as hydroxytamoxifen, the active metabolite of its parent drug tamoxifen, an antiestrogen that is inactive in its original form (Mürdter et al., 2011).

Using Drug-likeness analysis based on established criteria and calculated molecular descriptors, it is determined whether a substance is a candidate for further investigation as a potential drug. The obtained results indicate that all metabolites meet one of the most important criteria, Lipinski’s Rule of Five. This rule specifies that a substance should not have more than 5 hydrogen bond donors, no more than 10 hydrogen bond acceptors, a molecular weight below 500 Da, and a logP less than 5 (Lipinski et al., 2001). All tested compounds also meet other criteria, such as those related to the Veber Filter and QED Filter. However, metabolites obtained through the hydrolysis of gliclazide, specifically M7–M10, do not meet the Ghose Filter due to an insufficient number of atoms, which is one of the criteria. Since the hydroxylated metabolites M1–M6 satisfy the Ghose Filter criteria as well, they are suitable candidates for further analysis as potentially pharmacologically active substances. It is noteworthy that these hydroxylated metabolites M1-M6 are the ones that, according to the results of docking analysis, form the most stable complexes with the SUR1 receptor and show the highest affinity for this receptor.

In the VolSurf+ software package, molecular descriptors were determined to describe the physicochemical and pharmacokinetic properties of the investigated compounds. Considering that solubility is a limiting factor for the bioavailability of gliclazide (Biswal et al., 2009), it is interesting to note that all predicted metabolites exhibit better intrinsic solubility than gliclazide. However, the SOLY parameter cannot be considered the best indicator of a drug solubility in body fluids, as it describes the solubility of neutral species in water regardless of the pH environment. The PSA can be defined as the surface area of a molecule that contains highly electronegative atoms, primarily oxygen and nitrogen, including hydrogen atoms bonded to them. Based on the results, we see that the hydroxylated metabolites of gliclazide, M1–M6, have the largest PSA, i.e., those with an additional -OH group compared to gliclazide itself. It has been established that PSA shows a high correlation with intestinal absorption (Ertl et al., 2000). Unlike PSA, the HSA parameter represents the hydrophobic surface area of the molecule. It was found that all metabolites have lower HSA values than gliclazide. However, much better indicators of hydrophilic and hydrophobic properties are the PSAR and the PHSAR. All metabolites, except M8, have higher values for these two parameters compared to gliclazide. These results are consistent with the obtained logP values, showing that all investigated metabolites have lower logP values, indicating greater hydrophilicity than gliclazide. Since the optimal balance between solubility and permeability is found in substances with logP values in the range of 0–3 (Di and Kerns, 2003), according to the results of our *in silico* study, it may be anticipated that all metabolites, except for M9, are potential candidates for acceptable absorption and bioavailability. Based on the values of logP, PSA, PSAR, PHSAR, and SOLY, it is observed that the hydroxylated metabolites M1-M6 exhibit the most pronounced polar and hydrophilic properties, which could significantly contribute to their solubility in *in vivo* conditions. However, metabolites M2 and M6 show somewhat lower solubility compared to other hydroxylated derivatives, which could be explained by the different positions of the -OH group in the gliclazide molecule, contributing to different steric effects.

In addition to the mentioned physicochemical properties, potential drug candidates should also possess good pharmacokinetic properties. It is well documented that sulfonylureas bind tightly to plasma proteins which reduces their free concentration in plasma. Binding experiments conducted by Proks et al. showed that free concentration of gliclazide in the presence of human plasma was 15% (Proks et al., 2018). It should be noted that *in vivo* albumin glycation which is increased in in diabetes may have a direct impact on the binding ability towards gliclazide, and subsequently its free drug fraction and pharmacokinetics (Żurawska-Płaksej et al., 2018). However, it is observed that all metabolites have a lower percentage of plasma protein binding compared to gliclazide. Considering that only the free, unbound drug can exert its pharmacological effects (Mikov et al., 2017), it could be anticipated that the metabolites may have the potential for increased availability at the target site, which might enhance their pharmacological effects to some extent, though further investigation is needed to confirm this. These results are consistent with the VD parameter values, where it was found that all metabolites, except M1, have a higher VD, given that drugs with lower plasma protein binding generally have a higher VD (Burton et al., 2006). These data are consistent with experimentally obtained values, where the relatively low volume of distribution of gliclazide in healthy volunteers and patients (13–24 L) can also be partially explained by extensive plasma protein binding (85%–97%) (Sarkar et al., 2011).

Considering the lgBB parameter, the results show that metabolites M1-M6 have values lower than −50, indicating poor permeability through the blood-brain barrier. However, passage through the blood-brain barrier is not crucial from the pharmacodynamic standpoint of the observed drug, given that gliclazide exerts its pharmacological effect by binding to pancreatic cells (Bösenberg and van Zyl, 2008). Transport through Caco-2 cells for metabolites M1-M6 has been found to be lower than for gliclazide, but it does not differ significantly from gliclazide itself, which suggests good transport in *in vivo* conditions (Ðanić et al., 2019). The MetStab value above 50% for all observed compounds indicates stable behavior concerning metabolism by CYP3A4, which is of interest, since gliclazide is found to be metabolized by CYP2C9 and CYP3A4 (Kura et al., 2018).

The use of *in silico* methods to elucidate the drug-like properties and evaluate the therapeutic potential of gut microbiota-derived metabolites has been previously reported (Cañas et al., 2022; Mezhibovsky et al., 2023). To the best of our knowledge, this is the first study aimed to explore the pharmacological profile of gliclazide metabolites produced by gut microbiota. Apart from several aforementioned *in vitro* and *in vivo* studies (Al-Salami et al., 2008; Golocorbin-Kon et al., 2017; Ðanić et al., 2019), an extensive literature review has shown that this niche is still understudied and a more comprehensive insight into it is needed. While the results from a clinical interventional study in patients with T2DM have shown that gliclazide did not significantly alter fecal microbiome composition when used as add-on therapy in metformin-treated adults (van Bommel et al., 2020), there is no *in vivo* confirmation for the impact of intestinal microbiota on gliclazide metabolism so far. However, our research provides an important contribution to understanding how drugs like gliclazide may undergo microbial transformation, potentially affecting their therapeutic efficacy and safety. Given the increasing recognition of gut microbiota’s role in drug metabolism, our findings open up new avenues for further research in pharmacomicrobiomics. While the results provide valuable preliminary insights, we acknowledge that experimental studies are necessary to confirm the clinical relevance of these findings. Additionally, the specific microbial species involved in gliclazide metabolism were not identified, which limits the depth of understanding regarding which bacterial populations are responsible for the observed metabolic changes. Future studies should aim to address this gap by identifying the microbial communities involved.

## 5 Conclusion

The results of *in silico* analyses revealed significant differences in the physicochemical properties and potential pharmacokinetic profiles between gliclazide and its metabolic products produced by gut microbiota. Based on the results obtained from docking studies, aimed at finding compounds with the highest binding affinity for the target receptor, it was determined that the hydroxylated metabolites M1-M6 form more stable complexes compared to gliclazide, which opens the possibility of achieving an additional hypoglycemic effect mediated by the metabolites of the gut microbiota. Observing the results of the molecular descriptor analysis, it is noted that hydroxylated metabolites M1, M3, M4, and M5 possess the most favorable physicochemical and pharmacokinetic properties.

From the above, summarizing their physicochemical, pharmacokinetic, and pharmacodynamic properties, it can be concluded that among all compounds structurally related to gliclazide, hydroxylated metabolites M1, M3, M4, and M5, despite showing slightly lower predicted permeability through Caco-2 cells, represent the most suitable candidates with the most favorable pharmacological properties for further investigation. With the growing acknowledgment of gut microbiota influence on drug metabolism, our research paves the way for further exploration in the area of pharmacomicrobiomics. Although our study provides valuable preliminary insights, experimental research is necessary to confirm the clinical relevance of these results.

## Data Availability

The original contributions presented in the study are included in the article/supplementary material, further inquiries can be directed to the corresponding author.
